# Bilateral Bi-Cephalic Tdcs with Two Active Electrodes of the Same Polarity Modulates Bilateral Cognitive Processes Differentially

**DOI:** 10.1371/journal.pone.0071607

**Published:** 2013-08-08

**Authors:** Elise Klein, Anne Mann, Stefan Huber, Johannes Bloechle, Klaus Willmes, Ahmed A. Karim, Hans-Christoph Nuerk, Korbinian Moeller

**Affiliations:** 1 Knowledge Media Research Center, Neurocognition Lab, Tuebingen, Germany; 2 Section Neuropsychology, Department of Neurology, University Hospital, RWTH Aachen University, Aachen, Germany; 3 Brain Imaging Facility of the Interdisciplinary Centre for Clinical Research within the Faculty of Medicine at the RWTH Aachen University, Aachen, Germany; 4 Department of Psychology, Eberhard Karls University, Tuebingen, Germany; 5 Department of Psychiatry and Psychotherapy, Eberhard Karls University, Tuebingen, Germany; Hospital Nacional de Parapléjicos, Spain

## Abstract

Transcranial direct current stimulation (tDCS) is an innovative method to explore the causal structure-function relationship of brain areas. We investigated the specificity of bilateral bi-cephalic tDCS with two active electrodes of the same polarity (e.g., cathodal on both hemispheres) applied to intraparietal cortices bilaterally using a combined between- and within-task approach. Regarding between-task specificity, we observed that bilateral bi-cephalic tDCS affected a numerical (mental addition) but not a control task (colour word Stroop), indicating a specific influence of tDCS on numerical but not on domain general cognitive processes associated with the bilateral IPS. In particular, the numerical effect of distractor distance was more pronounced under cathodal than under anodal stimulation. Moreover, with respect to within-task specificity we only found the numerical distractor distance effect in mental addition to be modulated by direct current stimulation, whereas the effect of target identity was not affected. This implies a differential influence of bilateral bi-cephalic tDCS on the recruitment of different processing components within the same task (number magnitude processing vs. recognition of familiarity). In sum, this first successful application of bilateral bi-cephalic tDCS with two active electrodes of the same polarity in numerical cognition research corroborates the specific proposition of the Triple Code Model that number magnitude information is represented bilaterally in the intraparietal cortices.

## Introduction

In almost all instances when cognitive functions are investigated with functional Magnetic Resonance Imaging (fMRI), bilateral activation patterns are revealed. This is not only the case for numerical cognition [1], attention networks [2], [3], memory [4] or conscious processing [5]; but even for functions like language processing, which are commonly assumed to be lateralized, bilateral activation patterns are observed nevertheless (e.g., [6]; see [7] for a review). An innovative method to investigate bilateral processing is the use of transcranial direct current stimulation (tDCS), which will be described in more detail in the following.

### Transcranial Direct Current Stimulation and Cognitive Functions

Recent research has highlighted the importance of non-invasive brain stimulation such as tDCS as a means of modulating cognitive functioning via changing cortical excitability (e.g.[8]–[14]; see [15] for a review and discussion of future perspectives on tDCS). As a rule of thumb it is assumed that anodal tDCS increases cortical excitability, whereas cathodal tDCS decreases activity of the underlying brain tissue ( [16], see [17] for an overview; but see [18] for limitations and individual differences). Interestingly, excitability enhancing tDCS (e.g., by administering anodal stimulation, e.g., [17]) often seems to improve cognitive functioning. For instance, Fregni and colleagues [10] applied tDCS over the dorsolateral prefrontal cortex and observed enhanced working memory capacity.

However, as regards the evaluation of bilateral cognitive processing in numerical cognition, tDCS has been applied only as *bi-cephalic stimulation with different polarities;* this means that one pole (e.g., cathodal) was placed over the left hemisphere and the other pole (in this case, anodal stimulation) over the homologue area of the contralateral hemisphere (e.g., [13]; see [19] for a recent review). Such a stimulation design takes advantage of possible hemispheric asymmetries, for instance that one hemisphere is more involved in a particular cognitive process than the other hemisphere. However, because processing is facilitated in one hemisphere while being inhibited at the same time in the contralateral hemisphere, it is not possible to differentiate whether inhibition or excitation of either hemisphere modulated changes of a behavioral effect.

In contrast, *bilateral bi-cephalic tDCS with two active electrodes of the same polarity*, in which homologue structures in both hemispheres are stimulated with the same polarity (i.e., both anodal or both cathodal) offers the possibility to examine cognitive processing when both hemispheres and their connection contribute to the processing of a cognitive function or representation (e.g. [20]–[23]). Following above described rule of thumb, activation in both homologue areas should either be inhibited (by cathodal stimulation) or facilitated (by anodal stimulation). Consider the case that a cognitive process becomes slower and more error-prone after applying bilateral inhibition of certain homologue regions in both hemispheres. In this case, it can be suggested that either one or both homologue cortical regions is/are functionally necessary for the process at hand.

However, bilateral inhibition (or excitation) may have one important shortcoming. tDCS application is not very specific. For instance, the electrodes employed usually cover rather large areas (at least about 5 cm^2^ on the scalp). Moreover, stimulation extent, such as how deep and how wide the current actually stimulates the brain tissue, is still a matter of debate (see [19] for a recent review). Therefore, cognitive processes involved in the solution of many different cognitive tasks may be inhibited or facilitated rather than specific processes targeted. The serious impact of this problem is observed in patients with bilateral lesions. These patients are usually impaired in a large variety of tasks and processes because broad areas and their connections are damaged (e.g. [24]–[26]). Thereby, domain general cognitive processes like selective attention, alertness, and executive functioning are at least slowed down. This problem might also transfer to the case of bilateral bi-cephalic tDCS with two active electrodes of the same polarity, which is applied in the present study, because domain general cognitive processes subserved by a given cortical region may be inhibited/facilitated more strongly, when homologue areas in both hemispheres are inhibited or facilitated. Based on these considerations it may become obvious that while the application bilateral bi-cephalic tDCS with the same polarity may be a theoretically interesting concept, it is not yet clear whether it is possible to observe modulation of more specific cognitive processes such as differential effects in numerical cognition. In particular, while bilateral bi-cephalic tDCS has already been used previously [20]–[23], [27], [28], to the best of our knowledge there exists no study examining whether circumscribed numerical processes can be specifically modulated. So, there is no evidence for effects of bilateral bi-cephalic tDCS with the same polarity on processes of numerical cognitive processes in neurologically healthy participants. Therefore, identifying specific modulations induced by tDCS is a necessary requirement and will be addressed in the next paragraph.

### Between and Within-Task Indicators for Specificity of tDCS Application

Generally, a task specific influence of tDCS can only be evaluated in contrast to a viable control task. To this end, it is desirable to employ a domain general task serving as a control condition for unspecific DC stimulation effects. In such a between-task control approach, a typical test to explore application of bilateral bi-cephalic tDCS is administering a Stroop paradigm (e.g. [14]). The Stroop task is a widely used index of executive control [29], [30], which assesses the ability to inhibit a prepotent response as well as stimulus-driven control and response selection (e.g. [31]). At the neuro-functional level, the Stroop task is associated predominantly with activation of the anterior cingulate cortex and the dorsolateral prefrontal cortex with an additional involvement of bilateral intraparietal cortex (e.g. [32], [33]).

It is reasonable to employ a colour-word Stroop task in bilateral bi-cephalic tDCS with the same polarity as well, because the Stroop effect can be employed as a “marker” for an unspecific excitatory and/or inhibitory up- or down-regulation of parietal activation. If bilateral cathodal stimulation induces general down-regulation of parietal cognitive processes, the Stroop effect should be increased. Similarly, when bilateral anodal stimulation results in a general unspecific up-regulation of cognitive functions, the Stroop effect should be reduced.

However, cognitive tasks usually differ in more than one attribute of interest. So, while *between-task control* is adequate to control for the specificity of the *domain* of cognitive processing, it may not be adequate to address the specificity of the *representations* involved in cognitive processing within the same task (see, e.g. [34]). Therefore, *within-task* control allows for associating a specific effect with a certain representation, because within the same task differential tDCS influences indicate differential involvement of different representations. Yet, to instantiate within-task control, the task to be evaluated needs to draw on different representations. Additionally, for DC stimulation, the neural correlates of these representations should be known to put forward hypotheses regarding the effects of bilateral anodal or cathodal stimulation.

To address this issue, the case of number magnitude processing may be particularly informative. It is generally assumed that the processing of numerical magnitude and its hallmark effect, the distance effect (i.e., number magnitude comparison becomes more difficult as the numerical distance between numbers decreases e.g. [35]) is specifically associated with the bilateral intraparietal sulcus (IPS; [1] for an overview).

### Numerical Cognition as a Case of Specific Bilateral Parietal Processing

According to the most influential model of number processing, the Triple-Code Model of Dehaene and colleagues [1], [25], [36], numbers are represented in different formats within distinct cerebral areas. Most importantly, a bilateral parietal network around the IPS is supposed to be dedicated to the understanding and manipulation of numerical quantities. However, even though there are numerous brain imaging studies about the neural correlates of number processing (e.g. see [37], [38] for reviews; see [39] for a meta-analysis), functional magnetic resonance imaging (fMRI) and other imaging approaches cannot provide evidence whether the activation patterns observed for any numerical task are functionally necessary to perform this task or rather reflect some kind of co-activation of the respective cortex areas. Consequently, direct evidence for a causal structure-function relationship cannot be derived from imaging studies. So far, structure-function relationships have been investigated by either transcranial magnetic stimulation (TMS) studies, in which a virtual lesion is induced, or by lesion studies in brain-damaged patients. However, as regards the numerical magnitude representation, both of these approaches have important limitations. As the Triple Code Model [1], [25], [36] assumes number magnitude to be represented in intraparietal areas of both hemispheres, only a very specific bilateral parietal lesion (virtually as induced by TMS or permanently as found in patients) would lead to an impairment of number magnitude processing as indicated by deficient number comparison performance.

Nevertheless, employing repetitive *unilateral* TMS (rTMS), Knops et al. [40] were able to demonstrate the functional relevance of the IPS for number magnitude processing (see also [41]–[43]): A virtual lesion in the left IPS increased the numerical distance effect. Discriminability between numbers can thus be impaired by a virtual lesion in the left IPS (see also [44] for a comparable impact of unilateral TMS on mental arithmetic). Nevertheless, there is currently no study using TMS bilaterally.

On the other hand, so far evidence from lesion-based studies concerning the number magnitude representation was often confounded with additional pathologies such as in Gerstmann’s syndrome (e.g. [24]) or posterior cortical atrophy (e.g. [26]). Additionally, bilateral vascular infarction does usually not occur due to the rich vascularisation around the IPS. Therefore, the vast majority of lesion studies on number processing capabilities are single-case studies after unilateral brain damage (e.g.[45]–[47]; for a review see [48]). Thus, there is currently no report of a patient with bilateral damage confined to the IPS and its influence on number magnitude processing.

### The Present Study

#### tDCS application

So far, there are only few studies on the effect of tDCS on number magnitude processing. As described above, recent findings suggest functionally specific effects of bi-cephalic tDCS with two active electrodes of *different* polarity, which are apparent at the behavioural level [13]. The authors found that the polarity of brain stimulation (anodal/cathodal) specifically enhanced or impaired two indices of numerical proficiency. Both the acquisition of automatic number processing and the mapping of number onto space were enhanced by anodal and impaired by cathodal stimulation. This effect was specific for the processing of numerical symbols (i.e., not generalizing to a numerical control Stroop task). Furthermore and even more importantly, in the study of Cohen Kadosh et al. [13], the bilateral cortices were not inhibited or stimulated simultaneously. Therefore, it is not possible to differentiate whether inhibition or excitation of either hemisphere modulated the effect observed.

Differing from Cohen Kadosh et al. [13], we applied tDCS *bilateral bi-cephalic with two active electrodes of the same polarity* to further investigate the causal structure-function relationship between the bilateral IPS and number processing. The functional specificity of bilateral IPS was examined by systematically evaluating influences of cathodal and anodal tDC stimulation on performance in, both, within- and between-task approaches.

#### Within-task control: Different representations in a two-digit addition task

In an addition task participants had to indicate, which one of two solution probes was either the correct result (identical target, e.g., 25+31 = 56 or 51) or closest to the correct result (non-identical target, e.g., 25+31 = 54 or 51). The factors distractor distance (reflecting the distance between target and distractor) and target identity (reflecting the distance between the correct result and the target) were manipulated orthogonally (for a more detailed description of task and stimulus properties see [49]). The authors observed that decreasing values of distractor distance as well as non-identical targets were both associated with an increase of activation in areas subserving magnitude-related processing such as the posterior IPS, bilaterally. Therefore, tDCS was applied to the respective locations of these neural correlates on the scalp to identify possible specific effects due to bilateral bi-cephalic tDCS application with the same polarity.

However, there is an important difference between the effect of distractor distance and target identity, which may allow for within-task control: The effect of distractor distance seems to reflect a distance effect more purely than the effect of target identity. In the original study [49] stronger involvement of the IPS for distractor distance than for target identity was reflected by additional activation in the horizontal part of the IPS (hIPS). Importantly, no activation of the hIPS was observed for target identity. Since in 50% of the stimuli the target matches the correct result, processes such as recognizing familiar objects may be recruited in addition to number magnitude information. Therefore, one might expect a differential effect of bilateral unipolar tDCS on distractor distance and target identity.

#### Between-task control: colour word stroop

A colour word Stroop task was employed as between-task control for possible domain-general effects. While the Stroop task includes predominantly activation of the anterior cingulate cortex and the dorsolateral prefrontal cortex subserving executive control, it has been proposed that the bilateral IPS may be additionally involved when performing a Stroop task [31]. Along this vein, IPS activation in a colour word Stroop task was observed by Peterson and colleagues [33] at similar Talairach coordinates [−41–51 45] as observed for the numerical effect of distractor distance [−42–42 47] by Klein et al. [49].

### Hypotheses

The following hypotheses were derived based on the reported specific influence of anodal and cathodal stimulation on cognitive functions as well as on results from recent unilateral TMS studies.

1. First, in a between-task approach we aimed at investigating whether the DC stimulation effect is specific for number processing or a rather general effect. In case that the effect of tDC stimulation of parietal cortex areas is indeed number specific, the effect of stimulation on the mental addition task should be at least relatively stronger than on a colour word Stroop interference control task.
2. Second, in a within-task approach, differential effects of tDCS on distractor distance and target identity were expected.
  - If anodal stimulation facilitates cortical excitability and behavioural performance, the distractor distance effect should be reduced with bilateral bi-hemispheric anodal tDCS application. A reduced distance effect is taken to be indicative of increasing distinctiveness of the underlying representation of the single numbers’ magnitudes. In contrast, for target identity no modulation of the effect was expected.
  - On the other hand, it was hypothesized that the effect of distractor distance should increase under inhibitory bi-hemispheric cathodal stimulation,. This would be interpreted as decreasing distinctiveness and thus imply more overlap between the representation of the single numbers’ magnitudes. Again, for target identity no modulation was expected.
  - Importantly, independently of inhibitory or excitatory effects of cathodal and anodal stimulation, the size of the distractor distance effect should fall in between those observed for anodal and cathodal stimulation when sham stimulation is applied. Given this to be the case, we hypothesized that distance effects should differ significantly between anodal and cathodal stimulation at least.

## Methods

### Ethics Statement

Ethical approval was granted by the ethics committee of the Medical Faculty of the Eberhard Karls University Tuebingen and all procedures involved were in accordance with the latest version of the Declaration of Helsinki. All participants gave their written informed consent prior to the study.

### Participants

Twenty-four healthy volunteers, 23 right-handed, 1 left-handed, participated in the study (14 female; mean age 26.2 years, range 20–44 years). All participants did not have any neurological illness or serious medical condition and received 60 EUR as monetary compensation.

### Stimuli and Design

*Addition Task:* Stimulus material was based on the study by Klein and colleagues [49]. For the current study, this stimulus set was complemented by two additional sets, each of them matched for stimulus properties. Each set consisted of 192 different two-digit addition problems. For a detailed description of stimulus properties please refer to the methods section in Klein et al. [49]. Identical to Klein et al. [49], we manipulated the factors target identity and distractor distance. Targets were either identical or non-identical with the correct result. Target distances ranged from 0 (i.e., when target and correct result were identical such as in, e.g., 25+31 = 56 or 51 with the correct result and the target being 56) up to 3 (i.e., target and correct result were non-identical), while distractor distance ranged from 4 to 9 for small and from 14 to 19 for large distances between correct result and distractor. Thus, the experimental within-participant 3×2×2 design in the addition task comprised the factors stimulation condition (anodal vs. sham vs. cathodal), distractor distance (small vs. large distance between correct result and distractor), and target identity [0 (target identical with correct result) vs. larger than 0 (non-identical target)].

#### Colour-word stroop

In a computerized colour word Stroop task stimuli were colour words in different ink colours (yellow, red, green, and blue, respectively) presented in central position on the screen. Congruent stimuli were those in which colour word and ink colour were matched (e.g., ‘GREEN’ printed in green colour), while colour word and ink colour differed for incongruent stimuli (e.g., ‘GREEN’ printed in yellow).

### Procedure

**Figure 1:**
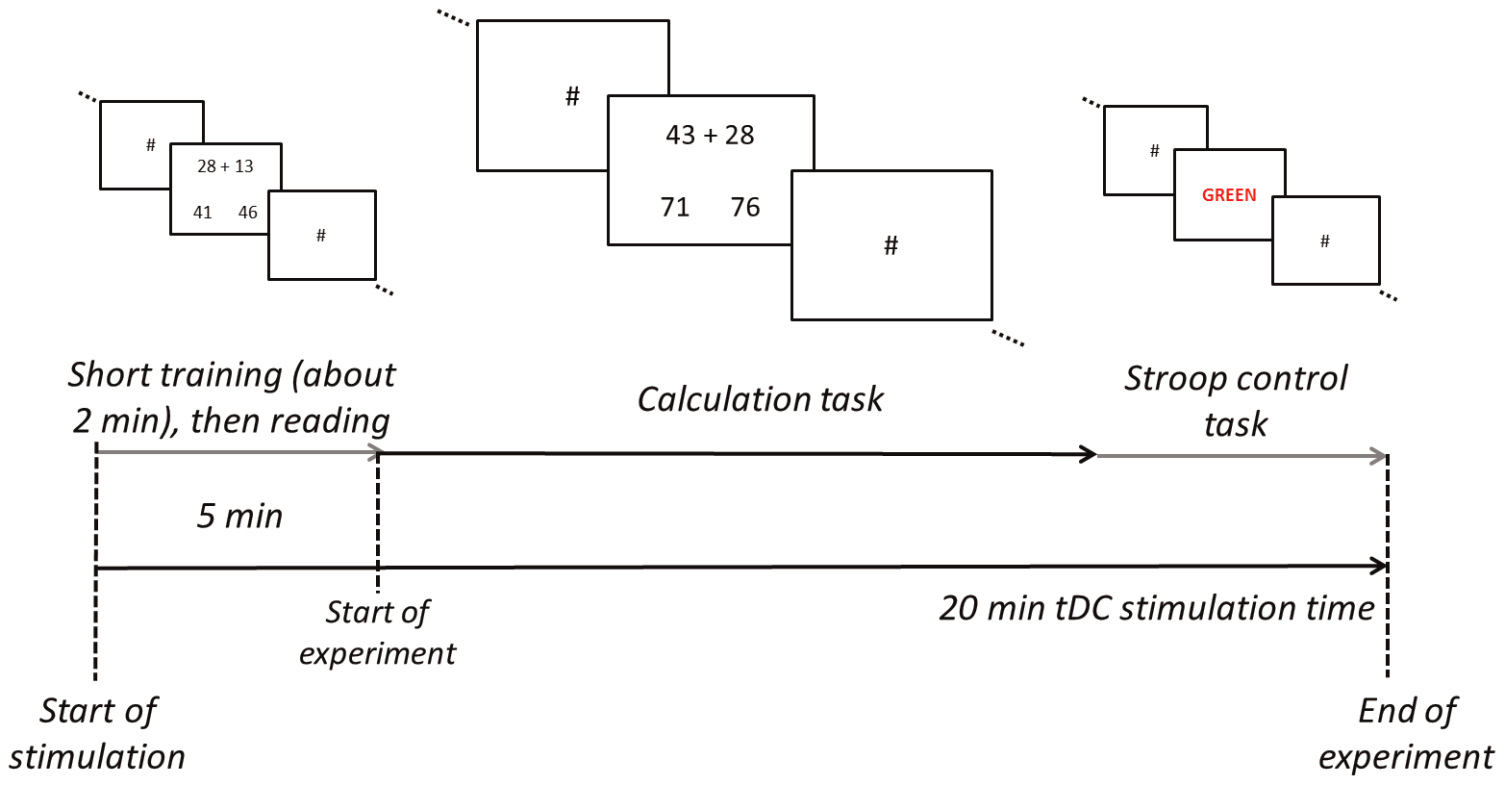
Schematic illustration of the experimental protocol.

To familiarize participants with the arithmetic task and the small tickling sensation on the scalp and to reduce potential training effects during tDCS data acquisition, in each session all volunteers solved 30 practice problems not included in the critical stimulus set before starting the actual experiment. In this initial training phase, the respective type of stimulation was administered to establish the tDCS effect already before starting the actual testing phase (see Figure 1). This aspect of the study design is important, since it has been repeatedly shown that tDCS application is not effective when administered for less than 30 sec [17]. To ensure comparable tDC stimulation for the critical items, direct current was applied throughout the whole experiment (max. 20 minutes) with a 5 min forerun to reach maximum effects (see [14] for a similar procedure; [50]). Importantly, the critical task was started after 5 min of tDC stimulation irrespective of the time taken by the practice items (all participants finished the training items within 3 min).

Each participant performed one anodal, one cathodal and one sham tDCS session. Session order was counterbalanced across participants with an interval of at least one week between sessions to avoid long-term stimulation effects [13]. The sessions lasted about 60 minutes each (including skin preparation, electrode placement, training phase, testing phase, and performing the control colour word Stroop task). Moreover, to avoid training effects, different stimulus sets (matched in all relevant stimulus properties) were used for each type of stimulation to avoid memory and learning effects.

Participants were seated approximately 50 cm from the screen in a dimly lit room. All stimuli were presented in white Arial font (size 26) against a black background using Presentation software (http://nbs.neuro-bs.com/presentation). In a choice-reaction paradigm, two-digit addition problems were presented centrally above a pair of solution probes in Arabic notation. Participants had to indicate the solution probe which was either identical (50% of the trials) or closest to the correct result (50% of the trials) as fast and as accurately as possible by pressing a corresponding button either with the left or the right hand. Each trial was preceded by a fixation cross presented for 500 ms. Each problem was presented until one of the response buttons was pressed or the time limit of 5 seconds was reached. Every 48 trials a short break of 15 seconds each was implemented. Trial order was pseudo-randomized.

In the control colour word Stroop task, participants were asked to identify the respective ink colour, in which the word was printed, by pressing adjacent colour-coded buttons on a standard QWERTZ keyboard (“v” for red, “b” for yellow, “n” for green, and “m” for blue). The task started with 24 practice items followed by 96 critical items with item order randomized. Each trial was preceded by a fixation cross presented for 500 ms. Following a blank screen presented for 500 ms, each item was shown until a button was pressed with an inter-trial interval of 1000 ms.

### tDCS Application

**Figure 2:**
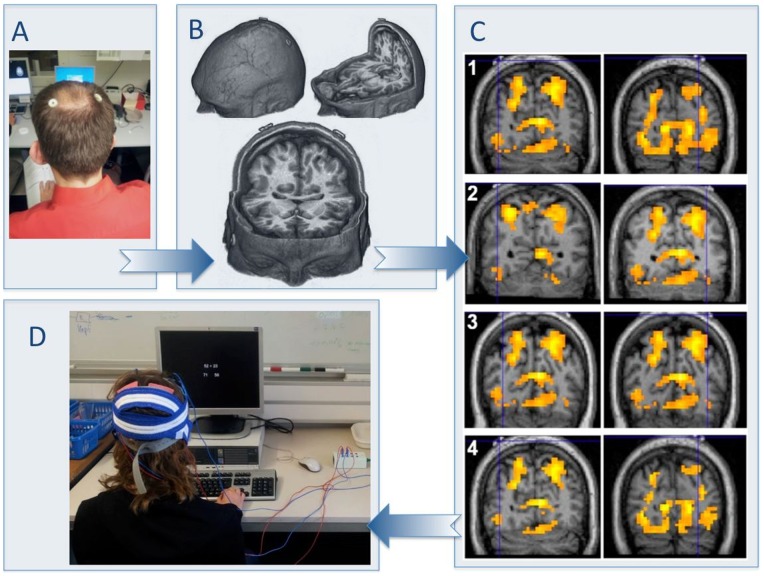
Determination of the parietal electrode positions and setup for the tDCS application. A: To validate whether positions P3 and P4 of the 10–20 international system for EEG electrode placement correspond to the bilateral IPS, external stereotactic fiducial markers were taped to the scalp of 4 participants at P3 and P4 before entering the scanner. B: Panel B reflects the anatomical validation of the correct position of the fiducial markers over the IPS. C: Panel C depicts functional validation of the correct position of the markers by displaying the original fMRI effects for target identity and distractor distance of the study by Klein and colleagues (2009) in the IPS on each of the individual normalized MRI scans. The blue crosshairs mark the centers of the fiducials. As can be seen, the electrode positions P3 and P4 correspond very closely to the localization of the activation peaks in the IPS observed by Klein et al. (2009). D: Setup of the tDCS application and presentation of the numerical task. The application of tDCS was transferred by two square scalp electrodes covered with conductive rubber (5×5 cm each) and red saline-soaked synthetic sponges over the target sites (P3 and P4) and two big reference electrodes (10×10 cm each, cf. the blue cables in Panel D) in the supra-orbital region.

In the fMRI study of Klein et al. [49] activations associated with the effects of distractor distance and target identity were observed for both, the bilateral IPS as well as the bilateral posterior IPS. To validate that the respective regions correspond to the positions P3 and P4 of the 10–20 international system for EEG electrode placement [51], additional MRI scans were acquired in 4 participants with external stereotaxic fiducial markers taped to the scalp at P3 and P4 before entering the scanner (Figure 2). The correct anatomical position of the fiducial markers over the IPS was validated using MRIcroGL software (http://www.mccauslandcenter.sc.edu/mricrogl/). In a second step, the correct position of the markers was functionally validated by depicting the original fMRI effects for target identity and distractor distance of the study by Klein and colleagues [49] on each of the individual normalized MRI scans using SPM8 software (http://www.fil.ion.ucl.ac.uk/spm/). As can be seen from Figure 2 (Panel B and C) the electrode positions P3 and P4 corresponded very closely to the localization of the activation peaks in the IPS observed by Klein et al. [49].

**Figure 3:**
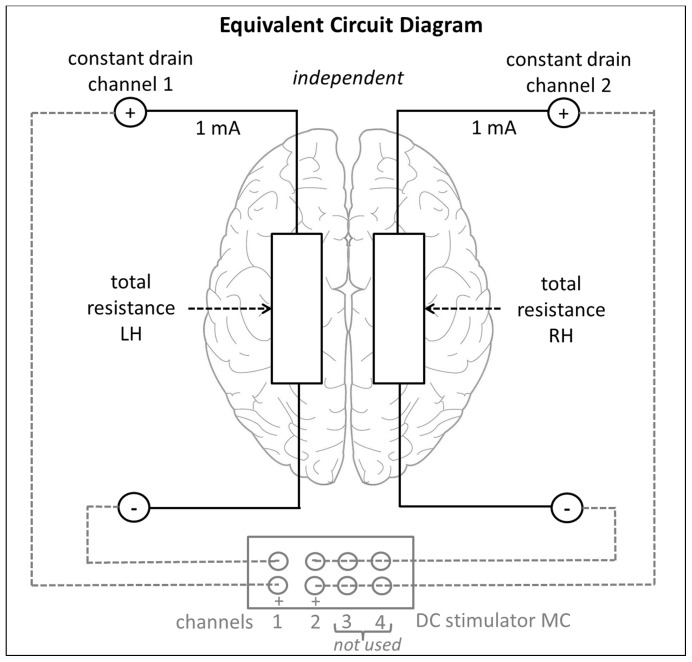
Schematic illustration of the tDCS set-up. As current flow through the.electrodes was regulated by two independent constant drain channels this system is linear. The DC-STIMULATOR MC provides freely programmable, micro-processor-controlled constant current sources using independent channels. By continuous (hardware- and software-based) monitoring of the electrode impedance it was ensured that the current path remains at the correct value for each hemisphere independently.

**Figure 4:**
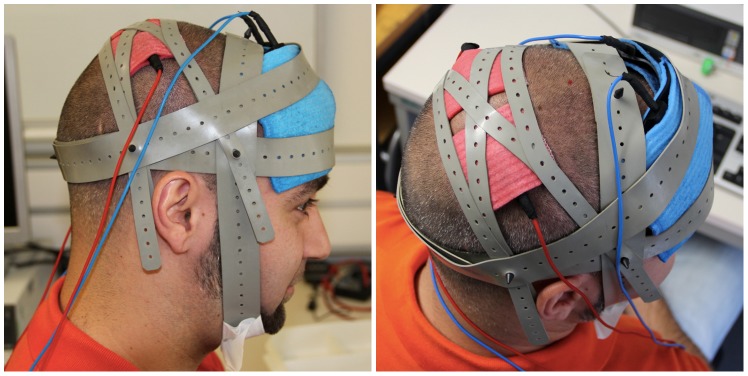
Photograph of the experimental tDCS montage used in the current study. The active small electrodes (5×5 cm^2^) are shown in red, the large reference electrodes in blue. Please note that the distance between active electrodes and reference electrodes is larger than 5 cm as recommended to minimize current flow through the skin (see [66] for a review).

During stimulation conditions, the application of tDCS was transferred by two square scalp electrodes covered with conductive rubber (5×5 cm each) and saline-soaked synthetic sponges over the target sites (P3 and P4) and two big reference electrodes (10×10 cm each) in the supra-orbital region. For optimal and safe stimulation of the target regions the DC-STIMULATOR MC by Neuroconn was used (Illmenau, Germany, http://www.neuroconn.de/dc-stimulator_mc_en/), which provides 8 freely programmable, micro-processor-controlled constant current sources using independent channels. By continuous (hardware- and software-based) monitoring of the electrode impedances it was ensured that the current path remains at the correct value for each hemisphere independently with only small deviations allowed. Otherwise stimulation was automatically terminated (see also Figures 3 and 4 for illustrations of the tDCS montage). Thus, current density and electrical resistance could be monitored separately for each of the two channels delivering current to the relevant regions P3 and P4. Therefore, a current of 1 mA each was applied to the target regions via two different channels resulting in a maximal current density of 0.04 mA/cm^2^ under the functionally active parietal electrodes. So, current density lies within the limits of recent safety protocols [17]. The total charge applied to the brain was 2.4 Coulomb and therefore 0.01% of the critical charge and within a range used in other tDCS studies (e.g. [52]).

At the beginning of the anodal and cathodal stimulation condition, current was increased slowly during the first 15 sec until the stimulation threshold of 1 mA for each of both channels was reached (ramp-up). At the end of stimulation current was decreased to 0 mA during the last 15 sec (ramp-down). Between ramp-up and ramp-down constant direct current (1 mA) was delivered for 20 minutes via each channel at the beginning of each session. In the sham condition, current was ramped-up during the first 15 sec as well, until the stimulation threshold was reached and constant current was delivered for 30 sec. Thereafter current was ramped down to 0 mA during 15 sec. This procedure ensured that in both stimulation and sham conditions participants experienced the initial itching that recedes over the first seconds of tDCS and made both conditions indistinguishable [53]. Interestingly, when asked which of the three sessions did not alter their ability to calculate (sham stimulation), none of the 24 participants was able to correctly identify this stimulation condition.

It is important to note that we choose the experimental set-up and montage with four cephalic electrodes intentionally to investigate our specific hypotheses regarding the processing of number magnitude information. Most authors of previous studies using bilateral bi-cephalic tDCS with the same polarity (e.g. [20], [27], [54], [55]) used extra-cephalic reference electrodes to ensure that brain tissue is not stimulated by the reference electrodes and to avoid confounding biases arising from electrodes with opposite polarities over the scalp.

**Figure 5:**
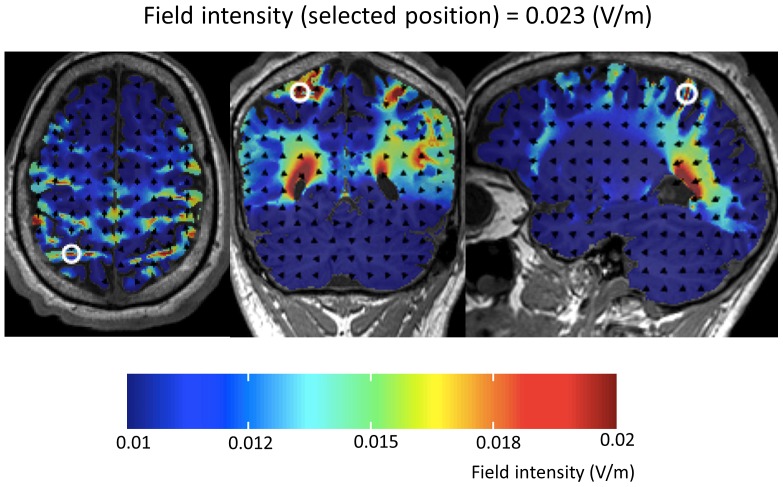
Results of the computer simulation of the experimental set-up as used in the present study. Please note that the resulting field intensity did not exceed 0.01 V/m in frontal brain areas but reached values equal or larger than 0.23 V/m in the bilateral intraparietal corices. According to Nitsche and Paulus [50] a minimum field intensity of 0.017 V/m is necessary to modify cortical excitability by tDCS in humans.

In the current study we used large reference electrodes (10×10 cm each) placed over frontal cortex regions (see Figure 4; please note that the person of the photograph has given written informed consent, as outlined in the PLOS consent form, to publication of their photograph). However, current density at our reference electrodes was far from being able to induce any cortical effect. Nitsche and Paulus [50] observed that a minimum current density of 0.017 mA/cm2 is necessary to modify cortical excitability by tDCS in humans. In particular, current density at our reference electrodes was maximally 0.01 mA/cm2 ( = 1 mA/10 cm×10 cm). Additionally, we ran a computer simulation of the tDCS montage employed in our study using HDExplore Software (v2.1, SOTERIX, http://www.soterixmedical.com/, see also [56]). This simulation software is specifically advocated for the DC-STIMULATOR MC by Neuroconn we used in the current study. As can be seen from the simulation results (Figure 5), the resulting field intensity indeed did not exceed 0.01 V/m in frontal brain areas. Therefore, we are confident that we neither stimulated the supra-orbital nor any further frontal region in a functionally relevant manner.

## Results

Analyses of reaction time (RT) were based on correct trials only resulting in a loss of 11.25% of the data for the addition and 3.96% for the Stroop task. Furthermore, response latencies smaller than 200 ms were not considered, and in a second step responses outside the interval of +/−3 standard deviations around the individual mean were excluded. An additional 0.83% and 2.63% of the data was excluded due to this trimming procedure for the addition and Stroop task, respectively.

### Within-task Stimulation Effects in Two-digit Addition

Effects of stimulation on participants’ processing of addition problems were analysed by a 3×2×2 within-participants analysis of variance (ANOVA) with the factors stimulation condition (anodal vs. sham vs. cathodal), distractor distance (small vs. large between correct result and distractor), and target identity (identical vs. non-identical target). Furthermore, we calculated linear contrast effects for the interactions between stimulation and distractor distance and between stimulation and target identity. If the type of stimulation differentially affects processing of magnitude related information as hypothesized, the effects of distractor distance and target identity should increase linearly from the anodal over sham to the cathodal stimulation condition.

The ANOVA revealed reliable main effects of target identity and distractor distance for RT data [target identity: *F*(1, 23) = 24.36, *p*<.001, *η_p_^2^* = .51; distractor distance: *F*(1, 23) = 46.86, *p*<001, *η_p_^2^* = .67]. Participants identified identical targets faster than non-identical targets (2479 ms vs. 2586 ms, respectively) and rejected distractors with large distances faster than those with small distances (2460 ms vs. 2605 ms, respectively; cf. Table 1). Furthermore, target identity significantly interacted with distractor distance [*F*(1, 23) = 5.38, *p*<05, *η_p_^2^* = .19] indicating that for non-identical targets the response difference between small and large distractor distances was larger than for identical targets [171 ms vs. 134 ms, respectively; *t*(24) = 2.08, *p*<.05]. All other main effects and interactions were not significant (all *F*s <1.7, all *p*s >.05).

**Table 1:** Mean response latencies for the three stimulation conditions in ms separately for the different distractor distance×target identity combinations.

		tDCS application		
Distractor distance	TargetIdentity	Anodal	Sham	Cathodal
small	identical	2539 (524)	2551 (460)	2534 (455)
	non-identical	2657 (546)	2660 (509)	2692 (488)
large	identical	2435 (474)	2410 (428)	2406 (446)
	non-identical	2504 (530)	2523 (451)	2482 (449)

Standard errors of the mean are given in parentheses (n = 24).

**Figure 6:**
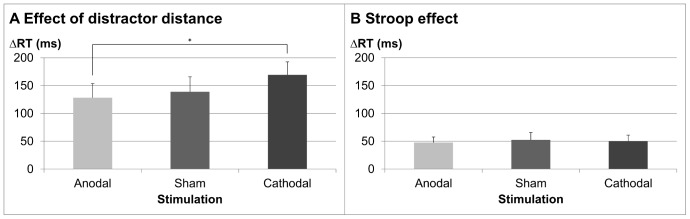
Effect of distractor distance and Stroop congruity separated for stimulation condition. Panel A reflects differences in RT between small and large distractor distances in the two digit addition task for the different stimulation conditions. The significant difference of the distractor distance effect between cathodal and anodal stimulation is marked by an asterisk. Panel B depicts RT differences between incongruent and congruent items in the colour word Stroop control task for the different stimulation conditions. Error bars reflect standard errors of the mean (SEM).

Most importantly, in accordance with our hypothesis we observed the linear interaction contrast for the interaction of distractor distance and stimulation condition was significant [*F*(1, 23) = 5.13, *p*<.05, *η_p_^2^* = .18]. As hypothesized, the distractor distance effect increased monotonically from the anodal stimulation condition followed by the sham and the cathodal stimulation condition (anodal: 128 ms, sham: 139 ms, and cathodal: 169 ms; see Figure 6). We also directly tested whether there was a smaller distractor distance effect under anodal as compared to cathodal stimulation. A significant *t*-test [*t*(23) = 2.27, *p*<.05] indicated that the distractor distance effect was indeed smaller in the anodal stimulation condition.

On the other hand, neither the linear interaction contrast between stimulation and target identity [*F*(1, 23) = 1.24, *p* = .28, *η_p_^2^* = .05] nor the direct comparison of target identity for anodal and cathodal stimulation [*t*(23) = 0.27, *p* = .39] reached significance. Please note that an identical ANOVA on error rates was significant for the main effect distractor distance [F(1, 23) = 76.57, p<.001, ηp^2^ = .77]. Participants made fewer errors when solving addition problems for large as compared to small distractor distances [7.60% vs. 14.90%].

Thus, as regards within-task effects, bilateral bi-cephalic tDCS with two active electrodes of the same polarity influenced the distractor distance effect assumed to be subserved by bilateral hIPS structures, but not the target identity effect, thus indicating within-task representation-specific effects of bilateral bi-cephalic tDCS with the same polarity.

### Between-task Control: The Colour Word Stroop Task

The impact of the different stimulation conditions on performance in a colour word Stroop task was assessed by a 3×2 within-participants ANOVA incorporating the factors stimulation condition (anodal vs. sham vs. cathodal) and congruency (congruent vs. incongruent for colour word and ink colour).

The ANOVA revealed a significant main effect of congruency [*F*(1, 23) = 22.03, *p*<.001, *η_p_^2^* = .54], indicating faster responses for congruent than for incongruent items (577 ms *vs.* 627 ms, respectively). The main effect of stimulation and the interaction between the stimulation and the congruency condition did not reach significance (both *F*s <1). Importantly, in contrast to the interaction between stimulation condition and distractor distance, there was no interaction between the linear stimulation trend component and congruency (*F* <1). Thus, we did not detect a significant differential effect of the tDCS conditions on performance in the colour word Stroop task. As for RT data, the congruency effect was significant indicating fewer errors for congruent than incongruent trials [3.39% vs. 4.38%; F(1, 23) = 9.06, p<.01, ηp^2^ = .28]. Neither the effect of tDC stimulation, nor the interaction with congruency were significant [Fs <1].

This between-task control indicates that the tDCS stimulation effect in the numerical task cannot be attributed exclusively to general task unspecific influences of bilateral unipolar tDCS affecting cognitive tasks independently of domain. In contrast, the differential effects between tasks indicate domain-specificity of tDCS effects.

## Discussion

In the current study we successfully applied *bilateral bi-cephalic tDCS with two active electrodes of the same polarity* with particular interest being paid to the specificity of this stimulation setting in a combined between- and within-task approach. Our observations were informative with respect to both aspects which will be discussed in turn.

### Between-task Specificity for Numerical Processing

We investigated whether bi-cephalic tDCS with two active electrodes of the same polarity applied to the intraparietal cortex bilaterally affected two tasks associated with intraparietal activation in a comparable or rather a differential manner. In particular, we aimed at evaluating whether the stimulation effect is specific to number processing or whether it rather represents a general effect of tDCS on cognitive processes assumed to be subserved by the bilateral IPS. Interestingly, the linear trend components for the stimulation by task interaction indicated that the influence of tDCS on cognitive processing was not only stronger for the numerical task but that there was no influence of tDCS on the colour word Stroop task at all. This between-task specificity is of particular interest, because previous patient data usually indicated multiple instead of specific impairments after brain damage to parietal cortices (e.g. [25], [26], [57]). Interestingly, it was not the case that in both tasks the more difficult condition was affected by tDCS. Instead, it was the more difficult condition of the numerical task only, which was specifically affected by bilateral bi-cephalic tDCS with two active electrodes of the same polarity. Thus, for the investigation and differentiation of cognitive functions associated with close or even overlapping cortex areas, bilateral bi-cephalic tDCS with the same polarity might even be more informative than patient studies, because most patients also suffer from general impairments of cognitive functions often obscuring differential effects on cognitive functioning.

However, this evidence for between-task specificity of bilateral bi-cephalic tDCS with same polarity was only part of the story, because we also observed within-task specificity.

### Within-task Specificity for Numerical Representations

We were specifically interested whether there are differential influences of bilateral bi-cephalic tDCS with two active electrodes of the same polarity on mental addition, shedding new light on the structure-function relationship between the bilateral intraparietal sulci and number magnitude processing in mental arithmetic. We hypothesized that the effect of distractor distance and the effect of target identity should be modulated differentially by anodal and cathodal tDC stimulation of the bilateral parietal cortex. As expected, we observed a significant linear increase in the distractor distance effect from bilateral anodal (excitatory) over sham to cathodal (inhibitory) tDCS with a significant difference between anodal and cathodal stimulation. This demonstrates that bilateral parietal application of tDCS can both support and inhibit the processing of number magnitude information. These findings add to recent TMS and lesion-based evidence suggesting a causal structure-function relationship in the human brain between bilateral intraparietal cortices and number processing. In line with our hypotheses, the effect of target identity was not modulated by the stimulation condition. This indicates that the specificity of bilateral bi-cephalic tDCS with the same polarity is not limited to differentiating between different tasks assumed to rely on shared cortex areas but also allows to differentiate between different representations involved in solving one task (here: a numerical task). While we observed an influence of tDCS on the processing of one representation (i.e., number magnitude) there was no effect on the other representations (i.e., recognizing familiarity).

### A Neuro-functional Account for within-task Dissociations

A possible explanation for the observed within-task dissociation may be found by inspecting the neuro-cognitive underpinnings of the two numerical effects of distractor and target identity more closely. First, the effect of distractor distance may be regarded as reflecting the reliance on and processing of numerical magnitude information more purely than the effect of target identity. Because in half of the trials target distance was zero and, thus, one of the two solution probes reflected the correct result, processes other than consideration of number magnitude information may be recruited to decide which one of the two solution probes is the target. For instance, it was shown that participants identified the target faster when it was identical to the correct result as compared to when it was the number closest to the correct result [49]. This suggests that whenever the target matches the correct result, the target may catch the participants’ eye faster, probably recruiting additional cortical areas such as the angular gyrus subserving the retrieval of arithmetic facts (e.g. [1]) and/or retrosplenial cortex (RC), which has been associated with the recognition of familiar objects and procedures (e.g. [58]; for a review see [59]). Such a RC activation was, for instance, observed by Klein et al. [60] in a similar mental addition task. In contrast, when the target does not match the correct solution (as for manipulation of distractor distance), participants cannot recruit similar additional recognition or retrieval processes but seem to rely more strongly on the processing of number magnitude information.

This argument of more specific reliance on number magnitude processing is further corroborated, when looking at the fMRI study by Klein et al. [49], from which the paradigm of the current study was taken. In the original fMRI data, distractor distance revealed not only overlapping activation with target identity in the bilateral posterior intraparietal cortices (e.g., in the left hemisphere at Talairach coordinates [−31, −66, 55]), but also additional intraparietal activation in the horizontal part of the IPS (hIPS; activation at [−42, −42, 47]). Relating this argument to the present tDCS study, it is very well conceivable that the current electric flow may have – due to the large electrodes placed in 5×5 cm^2^ over P3 and P4– may have affected the whole intraparietal cortex and, thus, the hIPS as well. Thereby, it may have enhanced (in anodal stimulation) or inhibited (in cathodal stimulation) the essential contribution of the hIPS to the solution of the addition problems in the distractor distance manipulation condition. Thereby, our data corroborate the propositions of the Triple Code Model (TCM, [1]) as regards the cortical representation of number magnitude information. In the next paragraph the implications of these results for our understanding of numerical cognition will be elaborated on.

### Number Magnitude Representation – Bilateral and Redundant?

One of the most important postulates of the TCM is that number magnitude information is represented bilaterally [1], [25], [36]. This means that only a bilateral parietal lesion (be it virtual or permanent) should lead to more or less complete impairment of the processing of quantitative numerical information. However, this proposition of a structure-function relationship cannot be tested reasonably by TMS due to practical reasons, because inhibitory and excitatory TMS are easy to discriminate for participants due to their different audible pulsing frequency. Therefore, it is not possible to apply excitatory and inhibitory TMS in a blinded fashion to participants in a within-participant design. Additionally, in patients the deficits of numerical processing were typically confounded with further systemic pathologies with bi-hemispheric pathologies (for a review see [48]).

In this study, we used bilateral bi-cephalic excitatory as well as inhibitory parietal tDCS with two active electrodes of the same polarity to investigate the structure-function relationship between the bilateral intraparietal cortex and number magnitude processing. We observed that bilateral anodal stimulation facilitated number magnitude processing as indicated by a reduced distractor distance effect whereas bilateral cathodal stimulation impeded number magnitude processing as indexed by an increase of the distractor distance effect. A decreased distractor distance effect can be interpreted as improvement in number magnitude processing, because it is assumed to indicate an increased discriminability of the numbers involved (in this case between the correct result and the distractor). Generally, the magnitude information of a number is supposed to be represented as a Gaussian distribution centred over the number, implying that neighbouring numbers are concomitantly activated with decreasing intensity the larger the distance to the currently processed number is (cf. [61]). This overlap between the representations of single numbers leads to a certain degree of uncertainty about the representation of a number’s magnitude. When the overlap decreases, the representations of the single numbers get more distinct and thus their discriminability increases. Thus, the respective numbers are easier to isolate. We suggest that anodal bilateral tDCS leads to a more distinct representation of number magnitude information, by reducing the overlap between the representations of the two probes involved in the addition task, reflected by a decreasing distractor distance effect. On the other hand, cathodal bilateral tDCS may have led to an increase of the overlap between the representations of the two probes making them harder to discriminate and thus the distractor distance effect increased.

Therefore, our data seem to corroborate the proposition of the TCM that the number magnitude representation seems to reside redundantly in both hemispheres. This is particularly interesting, because fMRI data repeatedly suggested number magnitude information to be represented bilaterally (and redundantly, see [39] for a recent meta-analysis). Yet, using fMRI one cannot decide, whether the observed activation indeed reflects a causal structure-function relationship between the bilateral intraparietal cortices and number magnitude processing. Thus, the current study is the first, investigating the structure-function relationship of bilateral IPS and number magnitude processing in a bilateral bi-cephalic approach with two electrodes of the same polarity, which indicated excitatory and inhibitory stimulation of the bilateral intraparietal cortex to modulate number processing reliably and specifically. Nevertheless, future studies are needed to substantiate this interpretation by complementing tDCS approaches using unilateral stimulation.

### Future Perspectives on tDCS to Investigate Neurocognitive Processes

The present study indicates that bilateral bi-cephalic tDCS with two active electrodes of the same polarity can be specifically informative regarding various research questions in numerical cognition. However, it is important to note that the present study is only a first step in investigating structure-function relationships using tDCS. So far, the current data are not informative regarding whether unilateral intraparietal stimulation of the bilateral intraparietal cortex can also modulate numerical performance. In particular so, as there is evidence in the literature that left and right intraparietal cortices may differentially contribute to numerical cognition (e.g. [40], [62], [63]). Therefore, in a next step, bi-cephalic tDCS studies with unilateral and bilateral stimulation would be highly desirable to investigate hemispheric specialization but also interaction more closely. For instance, hemispheric differences may be evaluated by contrasting the effects of bilateral and unilateral tDCS. When the effect of bilateral stimulation is comparable to the unilateral stimulation of one hemisphere whereas there is no effect of unilateral stimulation on the other hemisphere, this would indicate hemispheric specialization in the way that only the hemisphere affected by unilateral tDCS is functionally involved in the task at hand. Additionally, one might also think of comparing the effect of unilateral excitatory or inhibitory stimulation in a within-participant design with a functionally ineffective large electrode over the fronto-orbital scalp in a first step and/or, in a next step, contralateral reverse stimulation (e.g., cathodal stimulation of the left with anodal stimulation of the right hemisphere). In this way, differential contributions of the left/right intraparietal cortices to the task at hand could be systematically evaluated by comparing bilateral stimulation with simple unilateral stimulation and/or contralaterally opposing stimulation. Yet, future studies are needed to clarify whether such an all-or-nothing contribution of the two hemispheres is reasonable or whether lateralization within the human brain is better fitted by under- or overadditive relationships.

Furthermore, recently a new approach termed high definition tDCS (HD-tDCS) has been introduced. For HD-tDCS multiple (i.e., more than two) smaller sized gel electrodes are used to target specific cortical structures instead of using rather large and thus more or eless unspecific sponge electrodes. First studies using this new approach have been shown that HD-tDCS seems to induce more pronounced and longer lasting motor cortex excitability changes than sponge tDCS [64]. In the future this might be highly informative for numerical cognition research. In the current study we aimed at stimulating the intraparietal sulcus (mean length about 7 cm, see [65]) because the neural correlates of the three numerical effects, which we aimed at investigating (i.e., effects of target identity, distractor distance, and carry-over) were observed at different sites along the intraparietal sulcus within a range of about 5 cm (see [49]). Yet, it is well conceivable that future studies may be interested in more specific function-structure relationships such as for instance the functional influence of the hIPs on the processing of number magnitude or the influence of the angular gyrus on the retrieval of arithmetic facts (cf. [1] for a review). This would require much more focal tDC stimulation than provided by the sponge electrodes used in the current study and thus may call for the focal use of HD-tDCS.

Nevertheless, the specificity of bilateral bi-cephalic tDCS with two active electrodes of the same polarity observed in the present study points to a novel approach beyond patient studies to investigate the functional involvement and behavioural relevance of hemispheric specialization and interaction more closely in numerical cognition.

## Conclusions

In the present study, we investigated the specificity of bilateral bi-cephalic tDCS with two active electrodes of the same polarity in a combined between- and within-task approach, to further evaluate the causal structure-function relationship between the bilateral intraparietal cortices and number magnitude processing by stimulating the bilateral intraparietal cortex with transcranial direct current. Reflecting between-task specificity, we observed that bilateral bi-hemispheric tDCS with the same polarity affected a numerical (i.e., mental addition) but not a control task (i.e., colour word Stroop). This is interpreted as evidence for a specific influence of tDCS on numerical but not on more domain general cognitive processes associated with the IPS. Moreover, as regards within-task specificity, we found that excitatory stimulation reduced and inhibitory stimulation increased the numerical distractor distance effect in mental addition, whereas the effect of target identity was not affected by stimulation. This indicates that bilateral bi-bicphalic tDCS with the same polarity differentially affected different components within the same task (i.e., number magnitude information vs. recognizing familiarity). In summary, the successful employment of bilateral tDCS not only corroborates the proposition of the TCM by Dehaene and colleagues [1] that the number magnitude representation is situated in the bilateral intraparietal cortices, but also provides evidence for the potential of this experimental method in numerical cognition.
