# Correction: Bilateral Bi-Cephalic Tdcs with Two Active Electrodes of the Same Polarity Modulates Bilateral Cognitive Processes Differentially

**DOI:** 10.1371/annotation/c7fb111c-b586-4495-a55c-f779d1197810

**Published:** 2013-08-13

**Authors:** Elise Klein, Anne Mann, Stefan Huber, Johannes Bloechle, Klaus Willmes, Ahmed A. Karim, Hans-Christoph Nuerk, Korbinian Moeller

The current capitalization of letters in the term "tDCS" in the title is incorrect. The correct title is: "Bilateral Bi-Cephalic tDCS with Two Active Electrodes of the Same Polarity Modulates Bilateral Cognitive Processes Differentially." 

The correct citation is: Klein E, Mann A, Huber S, Bloechle J, Willmes K, et al. (2013) Bilateral Bi-Cephalic tDCS with Two Active Electrodes of the Same Polarity Modulates Bilateral Cognitive Processes Differentially. PLoS ONE 8(8): e71607. doi:10.1371/journal.pone.0071607. 

